# Wire-in-needle versus conventional syringe-on-needle technique for ultrasound-guided central venous catheter insertion in the internal jugular vein: the WIN randomized trial

**DOI:** 10.1007/s10877-024-01232-4

**Published:** 2024-10-14

**Authors:** Kristen K. Thomsen, Jovana Stekovic, Felix Köster, Alina Bergholz, Karim Kouz, Moritz Flick, Daniel I. Sessler, Christian Zöllner, Bernd Saugel, Leonie Schulte-Uentrop

**Affiliations:** 1 Department of Anaesthesiology, Center of Anaesthesiology and Intensive Care Medicine, University Medical Center Hamburg-Eppendorf, Hamburg, Germany; 2 Outcomes Research Consortium, Houston, Texas USA; 3 Center for Outcomes Research and Department of Anesthesiology, UTHealth, Houston, Texas USA

**Keywords:** Anesthesia, Complications, Guidewire, Procedural time, Surgery

## Abstract

**Purpose:**

There are different techniques for ultrasound-guided central venous catheter (CVC) insertion. When using the conventional syringe-on-needle technique, the syringe needs to be removed from the needle after venous puncture to pass the guidewire through the needle into the vein. When, alternatively, using the wire-in-needle technique, the needle is preloaded with the guidewire, and the guidewire—after venous puncture—is advanced into the vein under real-time ultrasound guidance. We tested the hypothesis that the wire-in-needle technique reduces the time to successful guidewire insertion in the internal jugular vein compared with the syringe-on-needle technique in adults.

**Methods:**

We randomized 250 patients to the wire-in-needle or syringe-on-needle technique. Our primary endpoint was the time to successful guidewire insertion in the internal jugular vein.

**Results:**

Two hundred and thirty eight patients were analyzed. The median (25th percentile, 75th percentile) time to successful guidewire insertion was 22 (16, 38) s in patients assigned to the wire-in-needle technique and 25 (19, 34) s in patients assigned to the syringe-on-needle technique (estimated location shift: 2 s; 95%-confidence-interval: − 1 to 5 s, *p* = 0.165). CVC insertion was successful on the first attempt in 103/116 patients (89%) assigned to the wire-in-needle technique and in 113/122 patients (93%) assigned to the syringe-on-needle technique. CVC insertion-related complications occurred in 8/116 patients (7%) assigned to the wire-in-needle technique and 19/122 patients (16%) assigned to the syringe-on-needle technique.

**Conclusion:**

The wire-in-needle technique—compared with the syringe-on-needle technique—did not reduce the time to successful guidewire insertion in the internal jugular vein. Clinicians can consider either technique for ultrasound-guided CVC insertion in adults.

## Introduction

Central venous catheters (CVCs) are commonly inserted in surgical or critically ill patients. CVC insertion can cause mechanical complications such as arterial puncture and hematoma [1, 2]. To reduce these complications, CVCs should routinely be inserted under real-time ultrasound guidance [2–6].

**Fig. 1:**
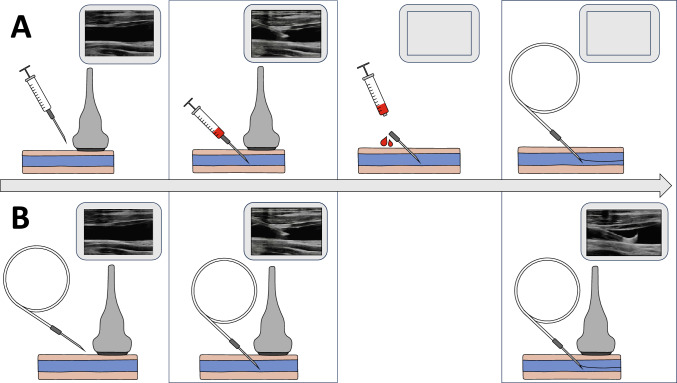
Illustration of the syringe-on-needle and wire-on-needle technique during ultrasound-guided central venous catheter insertion. **a** In patients assigned to the syringe-on-needle technique, a syringe was connected to the needle. The internal jugular vein was punctured under real-time ultrasound guidance. After the needle entered the internal jugular vein, blood was aspirated into the syringe. The operator then discontinued ultrasound guidance, disconnected the syringe from the needle, and advanced the guidewire through the needle in the internal jugular vein without ultrasound visualization. The correct guidewire position in the internal jugular vein was then confirmed with ultrasound. **b** In patients assigned to the wire-in-needle technique, the needle was preloaded with the guidewire and the internal jugular vein was punctured under real-time long-axis in-plane ultrasound guidance. After the needle entered the internal jugular vein, the guidewire was directly advanced through the needle into the vein under continuous ultrasound visualization. The correct guidewire position in the internal jugular vein was then confirmed with ultrasound

For conventional ultrasound-guided CVC insertion, a syringe is connected to the needle (syringe-on-needle technique) and the correct position of the needle tip in the target vein is confirmed by ultrasound visualization and aspiration of blood into the syringe (Fig. 1). After aspiration of blood, the operator must discontinue ultrasound guidance to remove the syringe from the needle and then pass the guidewire through the needle into the vein [3]. This step bears the risk of dislocating the needle tip and causing complications such as puncturing the posterior wall of the vein or an adjacent artery. An alternative ultrasound-guided CVC insertion approach is the syringe-free wire-in-needle technique (Fig. 1). Here, the needle is preloaded with the guidewire before the vein is punctured and the guidewire is advanced into the vein under real-time ultrasound guidance [7, 8].

It remains unknown which technique, wire-in-needle or conventional syringe-on-needle, provides better procedural quality and safety during ultrasound-guided CVC insertion. We, therefore, aimed to compare procedural time and complications of ultrasound-guided CVC insertion using the two techniques. Specifically, we tested the primary hypothesis that the wire-in-needle technique reduces the time to successful guidewire insertion in the internal jugular vein compared with the syringe-on-needle technique in adults. In addition, we evaluated whether the wire-in-needle technique improves the first-attempt success rate and reduces CVC insertion-related complications.

## Materials and method

### Study design and setting

This was a single-center randomized trial conducted in the Department of Anaesthesiology, Center of Anaesthesiology and Intensive Care Medicine, University Medical Center Hamburg-Eppendorf, Hamburg, Germany, between July 2022 and June 2023. The trial was approved by the local ethics committee (Ethikkommission der Ärztekammer Hamburg, Hamburg, Germany; registration number 2022–100852-BO-ff) on July 4th, 2022, and registered at ClinicalTrials.gov (NCT05452590) on July 6th, 2022. Participating patients provided written informed consent. We report the trial according to the CONSORT (Consolidated Standards of Reporting Trials) statement [9].

### Patient selection

We enrolled consenting patients ≥ 18 years scheduled for elective cardiac surgery in whom clinicians planned to insert a CVC in the internal jugular vein. We excluded patients in whom ultrasound visualization of the internal jugular vein in the long-axis (longitudinal) view was impossible because of anatomical reasons.

### Protocol

Patients had basic anesthetic monitoring (electrocardiogram, pulse oximetry, upper-arm cuff oscillometry). General anesthesia was induced using sufentanil, propofol or etomidate, and a neuromuscular blocking agent.

CVCs were inserted after anesthetic induction in a 15° Trendelenburg position under real-time ultrasound guidance in an internal jugular vein, preferably the right one. We used a triple-lumen CVC (Certofix ® Trio S 730, B. Braun, Melsungen, Germany) with an 18-gauge (1.02 mm diameter) introducer needle. The guidewire was 70 cm in length and 0.89 mm in diameter, with a guidewire advancer connectable to the syringe. Clinicians inserting CVCs were cardiac anesthesiologists with experience in both the wire-in-needle and the syringe-on-needle techniques.

Patients were randomized to ultrasound-guided CVC insertion using the wire-in-needle technique or to ultrasound-guided CVC insertion using the syringe-on-needle technique in a 1:1 ratio without blocking or stratification based on computer-generated codes. Group allocation was concealed with sequentially numbered opaque envelopes that were opened shortly before CVC insertion. Patients were blinded to group allocation, anesthesiologists naturally were not.

In patients assigned to the wire-in-needle technique, the needle was preloaded with the guidewire and the internal jugular vein was punctured under real-time long-axis in-plane ultrasound guidance (Fig. 1). After the needle entered the internal jugular vein, the guidewire was directly advanced through the needle into the vein under continuous real-time ultrasound visualization. The needle then was removed. The correct guidewire position in the internal jugular vein was then confirmed with ultrasound in both long-axis and short-axis views.

In patients assigned to the syringe-on-needle technique, a syringe was connected to the needle (Fig. 1). The internal jugular vein was punctured under real-time ultrasound guidance using either a long-axis in-plane or short-axis out-of-plane approach per clinicians’ preference. After the needle entered the internal jugular vein, blood was aspirated into the syringe. The operator then discontinued ultrasound guidance, disconnected the syringe from the needle, and advanced the guidewire through the needle in the internal jugular vein without ultrasound visualization. The needle then was removed. The correct guidewire position in the internal jugular vein was then confirmed with ultrasound in both long-axis and short-axis views.

### Endpoints

The primary endpoint was the time to successful guidewire insertion in the internal jugular vein—specifically, the cumulative time between skin puncture and removal of the needle after successful guidewire insertion. When multiple attempts were needed, we paused the time measurement when the needle was withdrawn and continued it again with the next skin puncture.

Secondary endpoints were the rate of successful CVC insertion at the first attempt; the number of attempts with each skin puncture being defined as a new attempt; the incidence of a collapsed composite outcome of CVC insertion-related complications (arterial puncture, posterior venous wall puncture or local hematoma, and cervical hematoma); the individual incidences of arterial puncture, posterior venous wall puncture or local hematoma, and cervical hematoma.

### Statistical analysis

Demographic and baseline characteristics are presented as absolute number (percentage) or median (25th percentile, 75th percentile).

We compared the primary endpoint (time to successful guidewire insertion in the internal jugular vein) between patients assigned to the wire-in-needle technique and patients assigned to the syringe-on-needle technique using a Mann–Whitney-*U* test—because it was not normally distributed (group-wise inspections of histograms). Secondary endpoints are presented as absolute number (percentage).

For the sample size calculation, we assumed that the time to successful guidewire insertion would have a standard deviation of 19 s [10]. A total of 238 patients (*n* = 119 patients per group) would provide 90% power at a 0.05 significance level to detect a difference in the time to successful guidewire insertion of at least 8 s. Allowing for 5% drop-outs, we planned to enroll 250 patients, 125 patients in each group.

## Results

**Fig. 2:**
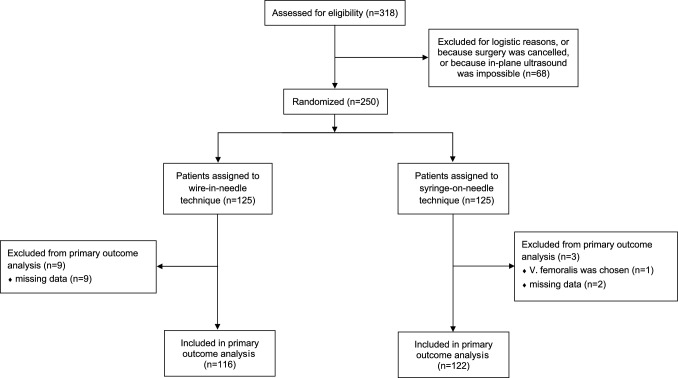
Flow chart illustrating randomization and reasons for exclusion

As planned, we randomized 250 patients. After randomization, we excluded 12 patients: 1 because the responsible clinician chose the femoral vein for CVC insertion and 11 because primary endpoint data were missing, usually because time to successful guidewire insertion was not recorded or recorded improperly. We thus finally analyzed 238 patients, with 116 patients assigned to the wire-in-needle technique and 122 patients assigned to the syringe-on-needle technique (Fig. 2). Patient characteristics are shown in Table 1.

**Table 1:** Patient characteristics

	Wire-in-needle technique (*n* = 116)	Syringe-on-needle technique (*n* = 122)
Age (years)	67 (59, 73)	66 (59, 72)
Height (cm)	176 (170, 182)	176 (170, 182)
Body mass index (kg m^−2^)	27 (24, 30)	26 (23, 29)
Female sex (*n*)	23 (20)	36 (30)

Categorical data are presented as absolute number (percentage), continuous data as median (25th percentile, 75th percentile)

**Fig. 3:**
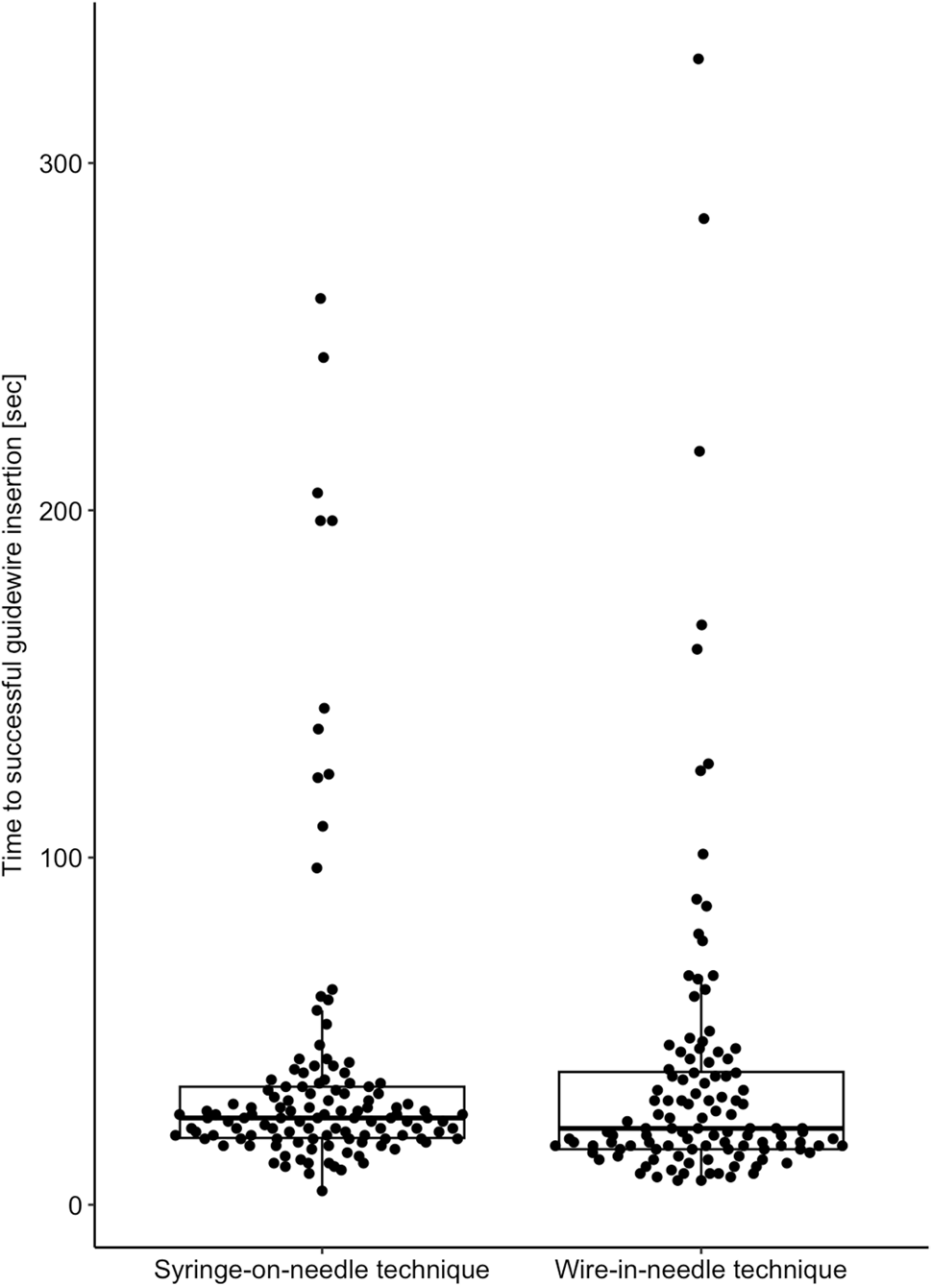
Boxplots with overlying one-dimensional scatter plot showing the time to successful guidewire insertion in patients assigned to the syringe-on-needle technique and the wire-in-needle technique. Boxes represent the 25th and 75th percentile and the range between them is the interquartile range. Inside the boxes, bold horizontal lines represent medians. The whiskers (extensions from the box) indicate the lowest and highest value no further than 1.5 times the interquartile range

The median (25th percentile, 75th percentile) time to successful guidewire insertion was 22 (16, 38) s in patients assigned to the wire-in-needle technique and 25 (19, 34) s in patients assigned to the syringe-on-needle technique (estimated location shift 2 s; 95% confidence interval − 1 to 5 s, *p* = 0.165) (Fig. 3).

CVC insertion was successful on the first attempt in 103 of 116 patients (89%) assigned to the wire-in-needle technique and in 113 of 122 patients (93%) assigned to the syringe-on-needle technique (Table 2). CVC insertion-related complications occurred in 8 of 116 patients (7%) assigned to the wire-in-needle technique and 19 of 122 patients (16%) assigned to the syringe-on-needle technique (Table 2).

**Table 2:** Central venous catheter insertion and complications

	Wire-in-needle technique (*n* = 116)	Syringe-on-needle technique (*n* = 122)
*Number of attempts for successful guidewire insertion*		
One attempt, *n*	103 (89)	113 (93)
Two attempts, *n*	12 (10)	7 (6)
Three attempts, *n*	0 (0)	1 (1)
Four attempts, *n*	1 (1)	0 (0)
*CVC insertion-related complications*		
Arterial puncture, *n*	0 (0)	1 (1)
Posterior venous wall puncture or local hematoma, *n*	8 (7)	18 (15)
Cervical hematoma, *n*	0 (0)	0 (0)

Data are presented as absolute number (percentage). CVC = central venous catheter

## Discussion

The wire-in-needle technique—compared with the syringe-on-needle technique—did not reduce the time to successful guidewire insertion in the internal jugular vein in adults. With both techniques, the first-attempt success rate was about 90%. However, the incidence of CVC insertion-related complications was halved when clinicians used the wire-in-needle technique (7 versus 16%).

A previous single-center trial compared the wire-in-needle and syringe-on-needle techniques in 80 adults having surgery [10]. In contrast to our results, this trial found that the wire-in-needle technique was faster (44 versus 55 s) and had a higher first-attempt success rate than the syringe-on-needle technique [10]. An important difference between the two trials is that we used a long-axis in-plane approach for the wire-in-needle technique whereas the previous trial used an oblique axis in-plane approach. Although the oblique axis approach allows simultaneous visualization of the vein, artery, and needle shaft with tip [11, 12], there is currently insufficient evidence that this approach is superior to long-axis approaches for ultrasound-guided CVC insertion [13, 14].

Our primary endpoint was the cumulative time to successful guidewire insertion which is clinically meaningful because numerous attempts and complications presumably increase the time to successful guidewire insertion. However, CVC insertion-related complications are far more important for patients, but are fortunately rare when real-time ultrasound guidance is used [15]. In a large prospective study, the incidence of mechanical complications during ultrasound-guided CVC insertion in the internal jugular vein was about 8% [15]. In our trial, the incidence of mechanical complications was 7% in patients assigned to the wire-in-needle technique and 16% in patients assigned to the syringe-on-needle technique. However, we also considered the minor and common complications posterior venous wall puncture and local hematoma. The wire-in-needle—compared with the syringe-on-needle—technique less often resulted in posterior wall puncture and hematoma, presumably because it does not require removing a syringe from the needle and thus reduces needle manipulation and allows continuous ultrasound-guidance. Whether the wire-in-needle technique can help reduce CVC insertion-related mechanical complications requires confirmation in a much larger trial.

With both techniques, the rate of successful CVC insertion at the first attempt was about 90%: 89% with the wire-in-needle and 93% with the syringe-on-needle technique. When using the wire-in-needle technique, the guidewire is advanced into the vein under real-time ultrasound guidance without aspiration of blood as a sign for successful cannulation. The correct position of the needle tip in the target vein therefore needs to be confirmed solely by ultrasound. The wire-in-needle technique thus requires good ultrasound skills, but the technique can be learned quickly and easily [8]. Additionally, the wire-in-needle technique does not allow ruling out accidental pleural injury by aspiration of air into the syringe. Pleural injury very rarely occurs during ultrasound-guided CVC insertion in the internal jugular vein [15]. We do not routinely use the wire-in-needle technique when inserting CVCs in the brachiocephalic or subclavian vein—where the risk of pleural injury is higher [15].

This—so far—is the largest trial comparing the wire-in-needle and the syringe-on-needle techniques. However, this was a single-center trial. Additionally, although we cannot quantify the pre-trial experience clinicians had with each technique, CVCs were inserted by cardiac anesthesiologists with ample experience in CVC insertion. Our results may thus not be generalizable to other institutions or clinical settings. Further, we only included patients having cardiac surgery who regularly have pleural drainage after surgery. We thus could not evaluate the incidence of CVC-related pneumothorax. In patients assigned to the syringe-on-needle technique, clinicians could use a long-axis/in-plane or short-axis/out-of-plane approach per their preference. Our results may thus have been influenced by the ultrasound approach clinicians chose in the syringe-on-needle group. As clinicians naturally chose the approach they were more comfortable with mandating a certain approach presumable would have resulted in a longer time to successful guidewire insertion in the syringe-on-needle group. Our trial was powered to detect differences in the time to successful guidewire insertion—but it was underpowered to detect differences in complication rates between the two groups. As complications caused by ultrasound-guided CVC insertion are rare, very large trials would be required to determine the effects of the wire-in-needle *versus* the syringe-on-needle technique on complications.

In conclusion, the wire-in-needle technique—compared with the syringe-on-needle technique—did not reduce the time to successful guidewire insertion in the internal jugular vein. Clinicians can thus consider either technique for ultrasound-guided CVC insertion in adults. Whether the wire-in-needle technique can help reduce CVC insertion-related mechanical complications requires confirmation in a much larger trial.

## Data Availability

Original data can be made available up on reasonable request.
